# Exploring Manifestations of TB-Related Stigma Experienced by Women in Kolkata, India

**DOI:** 10.29024/aogh.2383

**Published:** 2018-11-05

**Authors:** Reshmi Mukerji, Janet M. Turan

**Affiliations:** 1 Department of Microbiology, University of Alabama at Birmingham, Birmingham, AL, US; 2 Department of Health Care Organization and Policy, School of Public Health, University of Alabama at Birmingham, Birmingham, AL, US

## Abstract

**Background:**

Stigma associated with tuberculosis (TB) is still common in many societies, contributing to delays in treatment seeking and treatment non-compliance. India has the highest burden of TB in the world with female TB patients bearing a considerable burden of TB-related stigma.

**Objectives:**

This study aimed to explore the manifestations and consequences of stigma experienced by female TB patients in an urban setting in India and their strategies to cope with the social stigma of TB.

**Methods:**

Twenty qualitative interviews were conducted with female TB patients who were either currently on treatment or had undergone treatment at a TB clinic in Kolkata, India. Data were coded and analyzed with the NVivo qualitative software using a thematic approach.

**Results:**

Our results indicated that TB stigma mainly manifested through social isolation and avoidance due to fear of contagion, gossip and verbal abuse, failed marriage prospects, and neglect from family. Consequences of stigma described by the women included non-disclosure, feelings of guilt, and mental health issues including suicidal ideation. Positive coping strategies used by women to cope with the experiences of stigma included positive reframing, prayer, talking to other patients, focusing on school work, and relaxation activities. Negative coping activities included self-imposed social isolation and anger. In some cases, non-disclosure due to stigma had an impact on TB transmission and control behaviors.

**Conclusions:**

Stigma-reduction strategies, such as community awareness programs and formation of social support groups to dispel the myths and misconceptions associated with TB, may improve TB treatment seeking and adherence.

**Acknowledgement:**

Our deepest thanks to the Reverend, St. James’ Church, Dr. Ali Akbar Chowdhury (Medical Officer), staff and participants at the Calcutta Diocesan Tuberculosis Relief Trust, without whom this study would not be possible. We also thank Sushmita Mukherjee for help with translations. Lastly, we thank the Sparkman Center for Global Health at the University of Alabama at Birmingham for providing travel funds for this study.

## Introduction

India has one of the highest burdens of TB in the world, with about 2.2 million incident cases in 2016 [1]. Men have higher rates of TB than women due to a number of factors, including increased risk of infection and higher exposure rates [2]. However, women of reproductive age are known to have higher rates of disease progression and higher case fatality rates than men. Under-detection of TB among women could be attributed to reduced access to healthcare because of various social and economic factors [2,3,4,5].

One such factor that may impede a person’s ability to seek and adhere to TB treatment is stigma related to TB [2,6,7,8,9,10,11,12,13,14,15]. Stigma has been defined as the process by which an individual is devalued or discredited based on an undesirable attribute [16]. This form of social control is exercised by labeling, marginalizing, stereotyping and excluding the individual from broader society due to their undesirable traits. Some research suggests that the social stigma and fear associated with TB may impact women’s health behavior much more than men’s [2,6,17,18,19].

According to current conceptualizations, stigma is often classified into four categories: *perceived stigma* (feeling that those without the condition perceive people with the condition negatively), *anticipated stigma* (expectations of discrimination, stereotyping, and/or prejudice from others post disclosure due to a stigmatized condition or behavior), *enacted stigma* (experiences of discrimination, stereotyping, and/or prejudice from others in the past or present due to a stigmatizing condition or behavior), and *internalized stigma* (endorsing negative feelings and beliefs associated with the stigmatized condition and applying them to the self) [10,20]. There have been very few studies, to our knowledge, which have applied these dimensions of stigma to examine the behavioral and social consequences of TB [10,21].

Hence, this study seeks to address the research gaps around stigma faced by female TB patients in India through a detailed qualitative examination of the different dimensions of stigma. Furthermore, we examine how women cope with TB stigma, which has important implications for interventions. In order to understand these elements of TB stigma, we conducted in-depth qualitative interviews with twenty women patients at a Directly Observed Therapy-Short Course (DOTS)-TB clinic in urban India. We aim to use the study findings to inform TB control policies and programs to support women with TB in low-resource settings.

## Methods

### Study Area

This study was conducted in Kolkata, India, at a clinic run by the Calcutta Diocesan Tuberculosis Relief Trust, which provides DOTS-TB treatment to patients from the surrounding areas. The clinic is supported by the Calcutta Diocese of the Church of North India, and the Kolkata Municipal Corporation, Government of West Bengal, the latter being responsible for implementing all TB control activities in the state.

The clinic provides DOTS, which is a WHO-recommended treatment for TB where patients must take their medications while being directly observed [22]. At this clinic, patients presenting with TB symptoms must initially undergo sputum examination to get a bacteriological confirmation of TB. Then medications are provided free of cost and patients are directly observed taking their medication, either by a nurse or lay TB health workers. TB treatment generally lasts for six months but can continue up to nine months for some relapse cases. For the first two months of treatment (intensive phase) patients must come to the clinic three times a week and consume all medications under direct observation. For the final four months (continuation phase), patients come to the clinic once a week and collect their weekly medications. They must return empty blister packs the next week to get their next set of medicines.

### Study Population

Female TB patients over 18 years of age who were not pregnant were recruited for the study. Two categories of participants were recruited: (1) those who were currently in the six months of directly observed medication therapy (2) those who had completed treatment within the past six months or more.

Patients who came to the clinic to collect their medications, as well as those who had completed treatment but were on the clinic’s TB register, were contacted by clinic staff and the researcher if they met the inclusion criteria and asked to participate in an interview. All patients who agreed went through the informed consent process, with written informed consent being obtained. For those participants who could not read or write, the consent form was read out loud in the presence of a witness, and a thumb impression taken in lieu of a signature.

### Interviews

Interviews were carried out between May and June 2016. A sample size of twenty participants was chosen to ensure data saturation [23]. All participants were interviewed face-to-face at the clinic in the local languages (Hindi and Bengali) by a gender-matched interviewer, in a separate room, to ensure privacy. An in-depth interview guide was prepared to cover all topic areas of interest, although the interviewer had flexibility to explore additional questions. All of the interviews were audio recorded and notes were taken during the interviews to record non-verbal cues or emotions of the patient. After the first few interviews, the researcher went through the recordings and amended or modified parts of the interview guide to emphasize certain topic areas, come up with new probes, and adapt the questions to make it more suited to the population.

Participants were asked questions about their health-seeking behavior, medication experience, and barriers and facilitators to accessing care. In order to understand their experiences of stigma, participants were asked to describe their social interactions with family, friends, and community, as well as their experiences at this clinic and other health facilities since their TB diagnosis. Participants were asked to share stories or anecdotes they had heard about other TB patients. These questions were designed such that the responses captured the different dimensions of stigma (perceived, anticipated, enacted, and internalized) experienced by participants. The researcher used subtle probes to encourage areas of thought but refrained from giving any opinion of her own on any of the topics discussed. Interviews lasted from 30 minutes to one hour.

### Data Analysis

A thematic analysis approach was used to analyze the data, which involves examining data with a view to determining patterns within it. The “themes” are patterns that are present within the data and helps to describe phenomenon associated with the specific research questions. It also allows themes to emerge from the data, rather than using the data to answer specific questions the researcher had in mind [24].

The researcher listened to audio recordings and prepared brief reports after each interview to make sure all information and observations were recorded. This was followed by discussions with another investigator to make appropriate modifications. After the completion of all interviews, the audio recordings were transcribed and translated verbatim into English. All digital recording files and transcriptions were stored in a password-encrypted laptop and uploaded onto an encrypted server as a backup. Interviews were numbered in chronological order and patient names were not stored at any point. Basic demographic data collected from participants were stored in a password-protected file. All the transcripts were de-identified before being uploaded onto the server to ensure protection of participant privacy.

Once transcription was completed, the data were coded using NVivo qualitative coding software version 11.3.2. Broad codes were used first after which a layer of fine codes were applied to delineate the data further. Memos and annotations were used extensively throughout the coding process. Once the coding process was complete, the codes were combined into themes and an analytical report formulated based on the themes that emerged from the data.

### Ethical and Safety Considerations

The study protocol and consent procedure was approved by the Institutional Review Board, University of Alabama at Birmingham. Approval for the study was also obtained from the Medical Officer of the Calcutta Diocesan Tuberculosis Relief Trust, as well as a Member of the West Bengal Legislative Assembly. Participants who fulfilled the eligibility criteria and were interested in participating in the study were taken through the informed consent process during which they were told that participation was voluntary, the information would be strictly confidential, and it would not impact their medical care. They were also warned of the potential risks of participation, which included recounting painful memories. Participants who became emotional could take a break before continuing with the interview. One participant who reported a history of suicidal ideation was offered empathetic listening and resources at the nearest psychiatric hospital.

## Results

### Sample characteristics

Twenty in-depth interviews were conducted with women who were either currently on treatment or had previously completed treatment at a DOTS clinic. Given that the clinic was in a predominantly Muslim neighborhood, most of the women (80%) identified themselves as Muslim. Demographic characteristics of participants and their TB treatment status are described in further detail in Table 1.

**Table 1:** Sociodemographic characteristics of study participants attending DOTS-TB clinic in Kolkata, India.

Sample characteristics	Number (n = 20)	Percent

*Sociodemographics*		

Age group		
18–24 years	10	50
25–34 years	3	15
35–44 years	4	20
45–54 years	2	10
55–64 years	1	5
65 years and above	0	0
Religious Affiliation		
Muslim	16	80
Hindu	3	15
Christian	1	5
Marital Status		
Married	10	50
Single	10	50
Education Level		
No school	2	10
Primary	6	30
Secondary	9	45
Post-secondary	3	15
Employment status		
Housewife	6	30
Student	4	20
Employed/Informal work	6	30
None	4	20
Monthly household income		
Less than $100	14	78
$100–$200	4	22
*TB characteristics*		

Treatment status		
Currently on treatment	14	70
Completed treatment	6	30
Type of TB		
Pulmonary TB	14	70
MDR-TB	1	5
Extrapulmonary TB	5	25
Received TB treatment		
Once	13	65
Retreatment (more than once)	7	35

**Figure 1:**
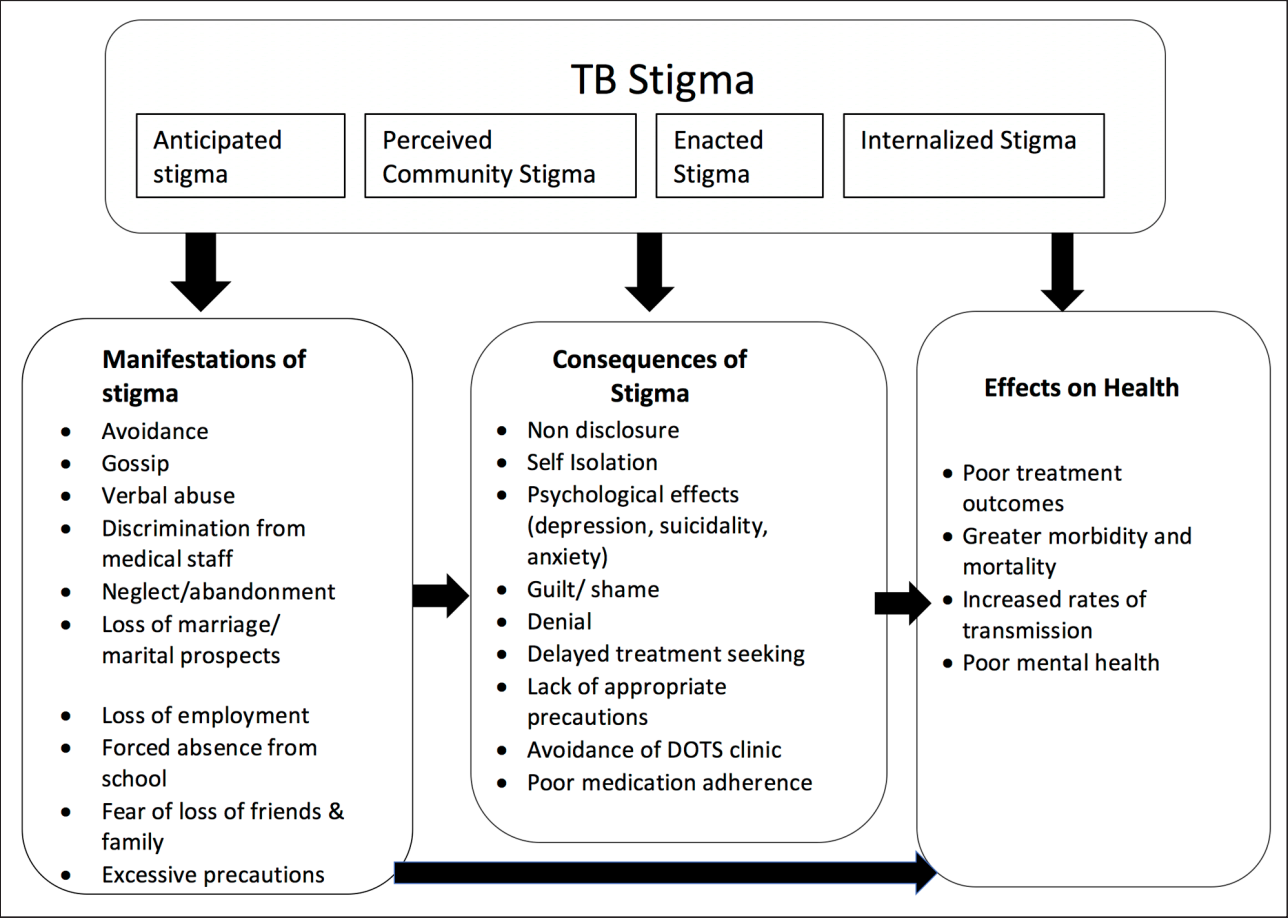
Conceptual framework of the dimensions of stigma, its manifestations and impact on women TB patients’ health outcomes (Adapted from Turan & Nyblade, 2013, [42]).

The data from these in-depth interviews were examined, and four main themes related to TB stigma emerged from our data: (1) manifestations of TB stigma: perceived, anticipated, and enacted, (2) consequences of fears and experiences of TB stigma, (3) ways in which women with TB cope with stigma, and (4) the potential impact of TB stigma on treatment and disease transmission. The conceptual framework that emerged from these analyses is presented in Figure 1.

### Theme One: Manifestations of stigma: Perceived, anticipated, and enacted stigma

#### Avoidance and excessive precautions due to fear of contagion

Participants perceived TB stigma to be present in their communities, due to the widespread fear of TB as a deadly infectious disease. People’s fear of TB caused them to avoid TB patients and take “appropriate precautions” such as sleeping and eating separately, keeping the patient’s belongings separate or refraining from sexual intercourse. Fear of contagion also extended to some healthcare settings where patients were asked to keep their distance from providers, and this was particularly distressing for patients:

“Everyone wants to avoid people like this. It happens because of fear of infection, like something dirty” (Patient 11, 21 years, single, currently on DOTS).

#### Gossip and verbal abuse

All participants who were interviewed in this clinic resided in the neighboring crowded slum communities where gossip was rampant, especially when a resident was known to have TB. One patient felt that women were the subjects of TB-related gossip more than men:

“When the man has it then there is less talk about it, but when it happens to women there is more talk because the woman does not really go outside much, and will stay at home more” (Patient 9, 30 years, married, currently on DOTS).

Several participants reported direct verbal abuse from neighbors or extended family. One patient complained about family members visiting her house, “as soon as they come they ask me to stay away, they just say it like that, it makes me so angry”. Another patient described a phone conversation with a nephew who lived abroad, “My nephew also told me, that if you talk to me on the phone then wipe the mouthpiece”. One participant described stories she had heard from a fellow patient, “[her] mother-in-law says, ‘don’t cough here, why did you cough here, get out!’ Her husband would say ‘put her in hospital, put her in hospital!’” There were also instances where neighbors would launch a direct verbal assault when a patient walked by:

“Like when I walk they will say, ‘hey you, you stay away from us’. It’s not like I’m walking close to them. If they see me coming, they will go into their house… in case I stop, they say ‘that TB infected one is coming’, they will go in saying, ‘she has come, she has come’…. they say, ‘look she has come, the witch, the one who devours her own home, ever since this one has been born she has been devouring her home’” (Patient 12, 20 years, single, currently on DOTS).

#### Loss of marriage or marital prospects and romantic relationships

The loss of marital prospects was the most commonly reported concern among younger, unmarried participants. Some participants reported that their marriage had been called off once the boy’s family found out they were sick. Others anticipated being mistreated after marriage because of their history of TB. Unmarried women also feared a relapse at the time of pregnancy, which they felt could possibly cause problems in their marriage:

“Mum worries a lot. If a match is fixed, and later then find out, then what will happen? There are a lot of illnesses that happen in life, what if someone has it after marriage, will they be thrown out? No, you have to keep them with you right. I also have to be careful that there is no neglect in what I eat or drink, so this disease does not come back. When people have babies, at the time of delivery, that time there are more problems. At that time, this disease can come. At that time there is weakness, it happens, and you have to be most careful at that time. That is what mum is saying. Feel very scared” (Patient 7, 20 years, single, currently on DOTS).

Participants also felt that women with TB were affected more than men in matters of marriage. As one participant explained that if a boy had TB he may still get married, but if a girl had TB the boy’s family would not want to fix marriage with her:

“The thing is if boys have it, *didi*, then people can ignore it. People are like yes, he had it, but he can get married…but with girls it’s like, she had it, so she can’t. This is how our mindset is like.” (Patient 7, 20 years, single, currently on DOTS).

Hence, many of the families were hesitant about disclosing participants’ TB status to the prospective grooms or their families. The interviews also suggested that younger, unmarried women tended to experience more stigma around marriage and relationships, as compared to older, married women:

“Yes, they will see young single women in a worse way; they will be like this girl had this thing. With old people, even if they have something, so what, their time is over anyway. They are married, they have children, now what” (Patient 14, 19 years, single, currently on DOTS).

#### Exclusion from school and work

Participants who were at school during their TB diagnosis did not disclose to school authorities because they felt sure that they would be asked to stay away. Participants who were working feared loss of employment once their TB status was known at work:

“I make some excuse up, like something came up at home so I need to go. Because if I tell them then they will throw me out of work…I am 90% sure” (Patient 11, 21 years, single, currently on DOTS).

#### Lack of support, neglect, or abandonment by family

Participants, particularly the married women, stated that they were responsible for all the housework in their homes. They had to cook, clean, wash clothes, and take the children to school despite their illness. One patient described how her family did not help with any housework even though she was sick and coughing, but at other times asked her to keep away because of her illness:

“When I cough then you guys never tell me to rest, but then you say you have disease you sit on one side” (Patient 1, 45 years, married, currently on DOTS).

### Theme Two: Consequences of fears and experiences of TB stigma

#### Lack of disclosure

Almost all the participants interviewed said that they did not disclose their illness to extended family, friends, or neighbors primarily due to anticipated stigma. However, several participants declared that their neighbors had found out about their illness because they had seen them getting medicines at the DOTS clinic:

“I think I should not tell them on my own, it should not come out of my mouth that I have this [illness]” (Patient 11, 21 years, single, currently on DOTS).

#### Feeling of guilt and diminished self-worth

Participants talked about having diminished self-worth and started blaming themselves for their illness after encountering avoidance or verbal abuse due to stigma:

“That reaction people have of ‘stay away from her, you will get infected from her’, you just say it, but you don’t think that the person who hears it feels really strange, feels really guilty that I have such a thing that everyone stays away from me” (Patient 8, 18 years, single, currently on DOTS).

#### Mental health issues: Depression, anxiety, and suicidal ideation

Several participants expressed shock and disbelief upon first hearing their diagnosis, since TB was a feared disease. The long treatment process caused patients to feel depressed. Unmarried participants described feeling “crazy” and anxious about how they would be treated after marriage. One patient was so upset with the experiences of constant verbal abuse from her neighbors that she had thoughts of killing herself:

“They say such things, so I mean I think, my mother has to listen to all this because of my illness, I think of taking a step, I mean I should not even say it. I think that it would be better to die. Then at least there would not be so many problems at home. When I hear those things, I think. When everyone says such things it really hurts me and I think it would have been better if I just died” (Patient 12, 20 years, single, currently on DOTS).

### Theme Three: Coping with or managing stigma

#### Positive coping mechanisms

Almost all of the participants interviewed mentioned that TB was a curable disease and some participants even explained that “TB had become normal these days” so there was no reason to be afraid or feel ashamed for having TB. All of the participants reiterated the importance of completing their treatment for a complete cure:

“This disease, TB, you know has become normal these days. Little children are getting it, adults are getting it, old people are getting it, everyone is getting it. But they all get cured” (Patient 1, 45 years, married, currently on DOTS).

Support from family and friends and medical staff were described as critical factors for participants to get through their experiences of TB. Participants described the benefits of talking to other patients at the clinic as it made them feel like they are not alone in their suffering:

“Yes, talking to others lightens the heart, the sadness goes away and you feel happier. You say something, and I’ll say something. It feels good, and the time passes by as well, as we get done taking the medication for the day” (Patient 15, 30 years, married, currently on DOTS).

Prayer was described as one of the biggest sources of strength for participants battling the negative experiences of TB. Relaxing activities such as listening to music and watching television were some other commonly reported coping mechanisms. Participants who were in school said that focusing on studies made them “feel very positive” and many expressed hopes of joining training programs to mitigate some of the stress of dealing with TB-related stigma.

#### Negative coping mechanisms

One of the most prevalent negative coping mechanisms reported was self-imposed social isolation. Participants cited several reasons for their isolation including feeling sick, as a precautionary measure to protect others, and anticipated stigma:

“So now I don’t talk to anyone, I stay at home, watch a little TV, I go to my mother’s house but take my handkerchief with me, now that I don’t have the cough, why should I cover my face and go. I just stay quiet and don’t talk to anyone” (Patient 1, 45 years, married, currently on DOTS).

A number of participants reported feeling more irritable since their diagnosis and described taking out their anger on their family members. Some participants described accepting their experiences of TB as their destiny, in a fatalistic way of dealing with their worries related to illness:

“I just remove the thoughts from my mind myself, I’m like what’s the point of thinking about all this, it’s in my destiny, you know how they say just accept. What is written in our destiny that will happen, no matter how much I think or how much you think we cannot change that. I think that and remove all the thoughts from my head and then talk to others, laugh with them” (Patient 9, 30 years, married, currently on DOTS).

### Theme Four: Effects of stigma on TB treatment and transmission

The potential effects of stigma on TB treatment and transmission were also explored in the interview data. In our study, participants described wasting months before getting proper treatment at DOTS clinics. Although non-disclosure was commonly practiced among all participants, some blamed their own infection on others who had kept their disease hidden:

“They all have it, but they all make excuses. They will say they don’t have it and I drank out of their cup, so I probably got it” (Patient 1, 45 years, married, currently on DOTS).

A number of participants revealed that some family members did not take precautions, in order to show love and support for the patient. Only one patient in this study wore a mask at the time of the interview (MDR-TB patient). She described her experience in the MDR-TB ward where family members would come in without masks: “She would never wear a mask because she loves her husband so much…she said ‘he has it and if I get it from him its ok, I’m not scared’”.

## Discussion

The historic perception of TB as killer infectious disease appears to be a primary driver of stigma associated with TB. In this study, participants reported on the manifestations of stigma that they had felt, feared, or experienced from extended family, friends, and neighbors.

As shown in previous studies, excessive precautions by the patient and their families continued long after their disease ceased to be infectious [9,25]. Patients also encountered verbal abuse from neighbors and subtle insults from extended family as has been shown in other settings [10,11,21]. There were also reports of negative behaviors from medical staff at some health facilities. This finding was of particular concern, as stigma from medical personnel acts as a deterrent to patients seeking treatment [11,26].

As seen in prior studies from South Asia, the loss of marital prospects and anticipation of mistreatment after marriage was a common theme among interviews with unmarried participants [6,19,27,28,29]. One of the reasons for this was the commonly held belief that TB would relapse at the time of pregnancy and could impact the unborn child [7,19,28]. Married women experienced stigma in the form of neglect from family members who expected them to continue in their traditional role of caregiver for the family despite illness. In India, women are responsible for all the household tasks, and the loss of physical strength due to TB contributes to the stigma experienced by these women [27,30,31].

The most important consequence of perceived and anticipated stigma was lack of disclosure [7,15,25,32]. However, participants reported that their neighbors usually found out about their illness due to the location of DOTS clinics, which are near their neighborhoods. This has important implications for DOTS, as patients may decide to avoid treatment for fear of being “discovered” by community members. The experience of TB-related stigma and the burden of who to disclose and who to hide the disease from is a stressful experience for the patient, resulting in depression and anxiety [30]. Negative experiences of stigma caused one study participant to contemplate suicide. One study from India reported suicidal ideation as an initial reaction to diagnosis of TB [27]. It has been shown that patients who internalize the experiences of stigma have feelings of guilt and diminished self-worth leading to poorer compliance to treatment [7,10,33,34]. Hence, patients would benefit by being counseled by clinic staff or trained mental health professionals throughout the treatment period, so that the mental health consequences of stigma can be lessened.

Coping mechanisms are means by which people mitigate some of the experiences of stigma. People with stronger coping mechanisms are known to have better treatment outcomes [35]. There are very few studies to date on the coping mechanisms used by TB patients to deal with their illness [33,35,36,37,38]. One study from India from the pre-DOTS era described that those who used coping mechanisms such as social isolation and prayer were more likely to be non-compliant [33]. All the patients in our study reported being compliant to treatment and hence we could not compare our results to these findings. However, contrary to their findings, many of our participants reported prayer as a source of strength and a helpful means to combat the negative experiences of TB. Patients also mentioned the benefits of talking to fellow patients at the DOTS clinic. Hence, the communal nature of DOTS clinics gives the additional benefits of social support while reducing feelings of social isolation, both which could have a positive impact on treatment adherence.

India’s national TB control program has done excellent work in spreading public messages about TB being a curable disease, which have helped allay some of the traditional fears associated with TB [39]. It has been shown that people who believe that TB is curable are less vulnerable to stigma and have better adherence [7,30]. In our sample, all participants used this positive reframing mechanism to cope with illness and stigma. Furthermore, our participants reported that the clinic staff were very caring and helped them complete treatment. Prior studies have shown that support from medical staff has a very positive impact on compliance and could be one of the reasons for such good compliance in our sample [33]. However, the possibility of selection bias cannot be discounted since all our participants were regulars at the clinic and were more likely to have more positive feelings towards it.

TB patients, especially women, have been known to delay treatment seeking by visiting private providers and traditional healers, for many reasons including TB stigma [6,14,15,40,41]. This treatment-seeking behavior means patients stay infectious for longer thus increasing the possibility of transmission to others. Furthermore, if patients do not disclose their illness due to anticipated stigma, they pose a risk to others who are in direct contact with them [15]. Hence, TB-related stigma has a direct impact on TB control and national TB control programs must incorporate stigma-reduction strategies in their future plans. TB clinics may benefit from specific stigma-reduction strategies, such as distributing information materials, seeking help from local religious leaders to dispel the myths associated with TB, forming “TB clubs” as a means of social support, or setting up income-generation programs for women living with TB [34,39].

Limitations of this study included a small sample size, which was restricted to one TB clinic in Kolkata, India. Hence, generalizing the study findings must be approached with caution. We were not able to include the experiences of men or of those women who had not sought treatment. However, women did share stories of their experiences prior to seeking treatment, and some of the reasons that they may have delayed seeking treatment. Further studies with patients from several TB clinics in the city may help validate the findings of this study.
